# Parental knowledge and barriers to cleft lip and palate care: a cross-cultural study from the Middle East and South Asia

**DOI:** 10.3389/fpubh.2025.1691553

**Published:** 2026-01-09

**Authors:** Sari M. Rabah, Heba Jafar Sabbagh, Alya AlZabin, Ebtesam Almajed, Razan Albrahim, Reema Aldawish, Lara Alyahiwi, Rand Alshabnan, Narmin M. Helal, Muhammad Asjad Khan, Muhammad Abrar Yousaf, Shaimaa Mohsen Refahee, Mohamed Mamdouh Koraitim, Mohammed Sadeq Kasem Albadani, Yousef Saleh Khader, Tamara Karim Al Halasa, Ola B. Al-Batayneh, Mohammad A. Albatayneh

**Affiliations:** 1 Plastic and Reconstructive Surgery Division, Department of Surgery, King Abdullah bin Abdulaziz University Hospital, Princess Nourah bint Abdulrahman University, Riyadh, Saudi Arabia; 2 Pediatric Dentistry Department, Faculty of Dentistry, King Abdulaziz University, Jeddah, Saudi Arabia; 3 College of Medicine, Princess Nourah bint Abdulrahman University, Riyadh, Saudi Arabia; 4 Department of Biological Sciences, Virtual University of Pakistan, Lahore, Pakistan; 5 Department of Neurosciences, Biomedicine and Movement Sciences, University of Verona, Verona, Italy; 6 Oral and Maxillofacial Surgery Department, Faculty of Dentistry, Fayoum University, Fayoum, Egypt; 7 Maxillofacial and Plastic Surgery Department, Faculty of Dentistry, Alexandria University, Alexandria, Egypt; 8 Cranio-Maxillofacial Department, Faculty of Dentistry, Alexandria University, Alexandria, Egypt; 9 Department of Public Health, Faculty of Medicine, Jordan University of Science and Technology, Irbid, Jordan; 10 Department of Preventive Dentistry, Faculty of Dentistry, Jordan University of Science and Technology, Irbid, Jordan; 11 Department of Orthodontics, Pediatric and Community Dentistry, College of Dental Medicine, University of Sharjah, Sharjah, United Arab Emirates; 12 Faculty of Medicine, Jordan University of Science and Technology, Irbid, Jordan

**Keywords:** cleft lip and palate, pediatrics, parental perception, quality of life, cleft lip, cleft palate, health policies, familial adjustment

## Abstract

**Background:**

Orofacial clefts, including cleft lip and/or palate (CLP), are common congenital anomalies that can cause feeding difficulties, speech articulation issues, dental problems, and ear complications. Timely treatment is essential to improve the quality of life for affected children. This study investigates parental knowledge, perceptions, and barriers to care for children with CLP across Saudi Arabia, Jordan, Egypt, and Pakistan. It explores cultural and systemic factors influencing healthcare-seeking behavior and offers recommendations to reduce cross-cultural disparities in awareness and access to care.

**Methods:**

A cross-sectional online survey targeted parents of children with CLP, recruited through hospital records and community support centers in the four countries. The questionnaire collected data on sociodemographics, parental knowledge, attitudes, practices regarding CLP, and barriers to accessing care.

**Results:**

A total of 505 parents participated, representing diverse regions of the Arab Gulf, North Africa, and South Asia. Most parents (91.9%) sought medical attention promptly after noticing CLP. However, knowledge varied: while many parents understood the medical nature of CLP, few recognized it as a syndrome or thought it was preventable. Travel and appointment barriers were prevalent; 70.3% traveled long distances for care, and 37.4% missed appointments due to work commitments.

**Conclusion:**

The findings reveal regional disparities in parental knowledge and access to care. Parents from higher socioeconomic backgrounds and the Arab Gulf reported better understanding and fewer barriers. Improving health education, transportation access, and community-based services is vital to support families and enhance CLP management.

## 1 Introduction

Cleft lip and/or palate (CLP) is the most prevalent congenital anomaly in the orofacial region. A cleft refers to an inborn gap or space in the upper lip, alveolus, or palate present at birth. CLP can occur independently or as part of a syndrome (1). Environmental factors, such as smoking, diabetes, alcohol use, and certain drugs, have been linked to CLP (2, 3). Children born with CLP require ongoing multidisciplinary care from birth through adulthood and are at higher risk of experiencing morbidity and mortality (4). Globally, CLP has a prevalence of approximately 0.45 per 1,000 live births, as estimated in a meta-analysis by Salari et al. (5) CLP significantly affects speech, hearing, appearance, and cognitive function, with long-term negative consequences for health and social integration (4). Results from a study indicated that children with oral clefts had higher healthcare utilization than their siblings without clefts, primarily due to hospital stays and medication intake related to congenital malformations (6). In a previous study by Soeselo et al. (7), it was found that many parents were unaware of CLP. A cross-sectional survey conducted among 200 women attending antenatal clinics in Nigeria found that only 19.8% had sufficient knowledge of CLP. Many respondents were unaware of the causes of CLP, the appropriate time for treatment, or whether the condition could be prevented (8). Understanding how individuals with CLP, their families, and their communities perceive the condition is crucial to the development of these children (9). Several studies have highlighted a high incidence of untreated clefts, suggesting that many parents lack awareness of the condition (10).

Given the crucial role parents play in their children's social and emotional growth, and the existing lack of comprehensive data on parental understanding of CLP, the primary objectives of this study are to assess parental knowledge about CLP, identify perceived barriers to care, and explore cultural attitudes in the selected countries. These countries were chosen to represent diverse cultural, social, and healthcare contexts within their regions: Saudi Arabia, Jordan, and Egypt each reflect distinct parts of the Middle East, while Pakistan represents South Asia. Each has unique cultural norms, healthcare infrastructure, and levels of awareness regarding congenital anomalies. By examining both the Middle East and South Asia, this study addresses a gap in the literature, as most prior research has focused on one region alone and has not explored the systemic challenges in each country that contribute to delays in treatment and care.

## 2 Materials and methods

### 2.1 Sample and data collection

Between June 2023 and June 2024, this research involved four countries: Saudi Arabia, Egypt, Jordan, and Pakistan. Participants were parents or legal guardians of children diagnosed with CLP and were recruited through hospital records and community support centers. This paper was written according to the Strengthening the Reporting of Observational Studies in Epidemiology (STROBE) Statement for reports of observational studies document (11). The study was conducted in accordance with the Declaration of Helsinki and was approved by the Institutional Review Boards of each country. All participants provided informed consent before the commencement of the study. Eligible participants were biological or legal parents/guardians of children with CLP aged ≥ 18 years. Participants were identified through multiple recruitment pathways, including patients followed at the principal investigator's institution, individuals listed in the national CLP registry, and parents participating in CLP community support groups who were invited to participate in the study. Due to the use of a snowball sampling approach, it was not feasible to determine an exact refusal rate. Sample size calculation using G^*^Power software (Version 3.1.9.6) for linear multiple regression (effect size 0.15, α = 0.05, power = 80%, three predictors) indicated a required sample size of 68 per country.

### 2.2 Survey instrument

A structured questionnaire of 44 items across seven sections was used for data collection: (I) Sociodemographic data: seven items addressing parental and child age, residence, parental education, and household income (HHI), (II) CLP description: six items exploring cleft characteristics, including type, family history, timing of diagnosis, relationship to the affected child, and associated anomalies, (III) CLP Knowledge: nine items assessing general knowledge regarding diagnosis, prognosis, risk factors, and prevention, selected questions were adapted from validated tools (8, 12), (IV) CLP Management: seven items evaluating awareness of treatment approaches, multidisciplinary care, complications, and prognosis. One question was adapted from a validated source (12), (V) Practice toward CLP: five items examining caregivers' practices, including timing of medical consultation, feeding methods, and prosthesis use, (VI) Attitude toward CLP: one validated item assessing caregiver attitudes (10), and (VII) Care barriers: nine items evaluating geographic, appointment, and scheduling barriers using a validated tool (13). The Cronbach's alpha for the nine items on care barriers was strong (α = 0.940). Questions were adapted from validated instruments where applicable, and the remaining items were developed by the authors and reviewed by two medical experts for content validity.

### 2.3 Statistical analysis

Descriptive statistics, including the mean and standard deviation for continuous variables, and frequencies and percentages for categorical variables, were applied. Multiple response dichotomy analysis was used for variables with more than one option. The Kolmogorov-Smirnov test and histograms were used to assess normality for metric variables. Cronbach's alpha was used to test the internal consistency and reliability of the questionnaires. The independent samples *t*-test assessed mean differences for metric variables across dichotomous variables, while the One-way ANOVA tested mean differences across categorical variables with more than two levels. Levene's test for equality of variance was used, and Welch's adjusted ANOVA was applied when variance inequality was noted. The chi-squared test of independence assessed correlations between categorical variables, and Pearson's correlation test was used for metric variables. The Likelihood Ratio-adjusted chi-squared test was preferred when necessary. Categorical factor analysis computed a socioeconomic index from parental income sufficiency, education level, and residential region. An exploratory factor analysis assessed the dimensionality of parental perceptions regarding medical appointments, access to services, treating physicians, and missed appointments. Multivariable generalized linear regression with Gamma distribution identified statistically significant predictors of parental perceptions of missed appointments and travel difficulties, expressed as adjusted risk rates with 95% confidence intervals. The Gamma distribution regression was preferred over conventional regression methods due to skewed multivariable residuals and over-dispersion. Multivariable linear regression was applied to analyze parents' knowledge of CLP and perceived difficulty accessing treating physicians, with beta coefficients and 95% confidence intervals. SPSS IBM version 28 was used for analysis, and statistical significance was considered at α = 0.05.

## 3 Results

A total of 505 parents of children with CLP participated in the study (Table 1). For comparative statistical analysis, data from participating countries were aggregated into three regional categories: Arab Gulf (*n* = 156), Middle East (*n* = 246), and Pakistan (*n* = 103) to ensure sufficient subgroup sizes and maintain statistical validity. Participants were from diverse regions, with 30.9% from the Arab Gulf, 48.7% from the Middle East, and 20.4% from Pakistan. The mean age of the children was 4.33 years (SD = 3.74). Mothers had a mean age of 32.86 years (SD = 7.41), while fathers had a mean age of 37.88 years (SD = 8.17). Educational attainment varied; 48.1% of mothers and 46.3% of fathers had a high school education or less, while 45.3% of mothers and 43.2% of fathers held university degrees. Regarding HHI, 19.4% reported being in debt, 38.6% met routine expenses, 30.7% could cover emergencies, and 11.3% had savings or investments. A family history of CLP was reported in 24.4% of cases, with most affected relatives being cousins, parents, or siblings. Regarding cleft type, 49.7% of children had no right-side CLP, 2.6% had an incomplete form, and 29.7% had a complete right-side CLP. On the left side, 45.9% were unaffected, while 22.6% had an incomplete form, and 31.5% had complete CLP. Palate involvement was present in 67.3%, with 27.7% having an incomplete cleft palate and 39.6% having a complete cleft palate. CLP was diagnosed prenatally in 21.4% of cases, and 25.5% of children had associated anomalies, primarily hearing, cardiac, or cognitive impairments.

**Table 1:** Sociodemographic characteristics, *N* = 505.

**Variable**	**Frequency**	**Percentage**
**Region**		
Arab Gulf State	156	30.9
Middle east	246	48.7
Pakistan	103	20.4
Affected child age (years), mean (SD)		4.33 (3.74)
**Child age group**		
< 1 year	80	15.8
1–3 years	191	37.8
4–6 years	107	21.2
7–0 years	86	17
≥11 years	41	8.1
Mother's age (years), mean (SD)		32.86 (7.41)
15.6-2.2,-1.3242ptFather's age (years), mean (SD)		37.88 (8.18)
**Mother's educational level**		
High school or less	243	48.1
University degree	229	45.3
Post-graduate degree	33	6.5
Father's educational level		
High school or less	234	46.3
University degree	218	43.2
15.6-2.2,-1.3242ptPost-graduate degree	53	10.5
**Household monthly income**		
In-debt	98	19.4
Just meet routine expenses	195	38.6
Meet routine expenses and emergency	155	30.7
31-2.2,-1.3242ptAble to save/invest money	57	11.3
**Descriptive analysis of the children's CLP medical history**		
**Do you have any other family member with CLP?**		
No	382	75.6
15.6-2.2,-1.3242ptYes	123	24.4
**What is the relationship of this CLP affected person to the child?**		
Uncle/aunt	12	9.8
Cousin	47	38.2
Sibling	28	22.8
Parent	33	26.8
15.6-2.2,-1.3242ptGrandparent	10	8.1
**Child's right side CLP type**		
Not affected	251	49.7
Incomplete	104	20.6
Complete	150	29.7
Not affected	232	45.9
Incomplete	114	22.6
15.6-2.2,-1.3242ptComplete	159	31.5
**What type of cleft palate does your child have?**		
Not affected	165	32.7
Incomplete	140	27.7
15.6-2.2,-1.3242ptComplete	200	39.6
**Was the cleft diagnosed during pregnancy?**		
No	397	78.6
15.6-2.2,-1.3242ptYes	108	21.4
**Does the child have any associated anomalies?**		
No	341	67.5
I don't know	35	6.9
15.6-2.2,-1.3242ptYes	129	25.5
**If the child had associated anomalies, what are they? (*****n*** = **129)**		
Congenital cardiac disability	40	31
Hearing disability	60	46.5
Cognitive disability	26	20.2
Mental disability	31	24
Other problems	11	8.5
Speech difficulty	2	1.6
Dental problems	3	2.3
Esophageal anomalies	1	0.8
15.6-2.2,-1.3242ptDifficulty breathing	4	3.1
**CLP sources of information (*****n*** = **500)**		
Internet	202	40
Social media	103	20.4
Hospital	268	53.1
Family member	46	9.1
School	11	2.2
15.6-2.2,-1.3242ptCausal meeting	11	2.2
**When was the first time you heard about CLP?**		
During the pregnancy	192	38
After having a child with CL/C	313	62

Countries were grouped into three regions (Arab Gulf, Middle East, and Pakistan) prior to analysis to maintain adequate sample size and statistical validity. Individual country-level data were not analyzed separately due to small sample sizes in some nations.

Table 2 details parental knowledge of CLP. While 64% correctly defined cleft palate, only 16.8% recognized CLP as a syndrome. Most parents (51.9%) acknowledged the possibility of congenital anomalies, and 48.9% understood the potential of prenatal diagnosis. However, only 22.2% believed CLP could be prevented. The correctly identified risk factors included chemical exposure (39.5%), consanguinity (31.7%), nicotine exposure (41.3%), teratogenic medications (59.4%), genetic predisposition (23.5%), maternal illness (56.6%), stress (56.9%), and inadequate folic acid intake (39.9%). Parental understanding of multidisciplinary care was generally high, with 70.6% recognizing orthodontists, 68.1% recognizing social workers, 95.8% identifying otolaryngologists, 68.1% identifying speech therapists, 95.6% recognizing plastic surgeons, and 95.8% identifying feeding specialists. Surgery was recognized as a primary treatment by 97.8%, while only 18.4% considered prostheses as an option.

**Table 2:** Descriptive analysis of the parental knowledge of CLP.

**Variable**	**Frequency**	**Percentage**
**What is cleft?**		
Opening of lips only	101	20
Opening on the lips and hard palate	323	64
Opening on the hard palate only	54	10.7
15.6-2.2,-1.3242ptI don't know	27	5.3
**Could CLP be syndromic or not?**	
No	147	29.1
I don't know	273	54.1
15.6-2.2,-1.3242ptYes	85	16.8
**Could CLP have other associated anomalies or not?**		
No	86	17
I don't know	157	31.1
15.6-2.2,-1.3242ptYes	262	51.9
**Can CLP be diagnosed before the child is born?**		
No	114	22.6
I don't know	144	28.5
15.6-2.2,-1.3242ptYes	247	48.9
**Can we prevent CLP?**		
No	157	31.1
I don't know	236	46.7
15.6-2.2,-1.3242ptYes	112	22.2
**Which of the following is a known CLP risk factors?** ***N*** = **281**		
Chemical substance exposure during pregnancy	111	39.5
Consanguineous marriage	89	31.7
Nicotine exposure	116	41.3
Medicines like phenytoin, valproic acid, trimethadione	167	59.4
Genetic	66	23.5
Maternal illness	159	56.6
Stress	160	56.9
Inadequate Intake Folic acid during pregnancy	112	39.9
**Does the management require a multidisciplinary approach?**		
No	27	5.3
I don't know	36	7.1
15.6-2.2,-1.3242ptYes	442	87.5
**Who are the participants of the team?**		
Prosthodontist	356	70.6
Orthodontist	356	70.6
Social workers	343	68.1
Otolaryngologist	483	95.8
Pedodentists	356	70.6
Speech therapist	343	68.1
Geneticist	343	68.1
Plastic/craniofacial surgeon	482	95.6
Feeding specialist	483	95.8
15.6-2.2,-1.3242ptNurse coordinator	482	95.6
**Is surgery a known treatment for CLP?**		
No	11	2.2
15.6-2.2,-1.3242ptYes	494	97.8
**Is Prosthesis a known treatment for CLP?**		
No	244	48.3
I don't know	168	33.3
15.6-2.2,-1.3242ptYes	93	18.4
**Are medications part of treatments for the CLP?**		
No	295	58.4
I don't know	132	26.1
Yes	78	15.4

Table 3 summarizes parental practices. Most parents (91.9%) sought medical attention immediately upon noticing CLP, while 5.3% did not seek any. Feeding practices included formula (57.8%), breastfeeding (15.2%), and both (26.9%), with 49.3% of parents using an upright feeding position (90-degree angle). A majority (63.2%) used a feeding prosthesis.

**Table 3:** Descriptive analysis of parental practices regarding CLP affected children.

**Variable**	**Frequency**	**Percentage**
**When did you seek medical attention?**		
When we noticed immediately	464	91.9
We waited a while	14	2.8
30-2.2,-9242ptWe didn't seek medical attention	27	5.3
**If you choose “waited a while” for the previous question, for**		
**how long have you waited?**		
2–8 weeks or less	3	21.4
6–12 Months	7	50
29.5-2.2,-1.3242pt>1 years	4	28.6
**What type of feeding does your child mainly depend on?**		
**(If applicable)**		
Formula feeding	292	57.8
Breastfeeding	77	15.2
Both Formula and Breast feeding	136	26.9
**Which position do you use while feeding your child?**		
Supine	69	13.7
45 Degrees	187	37
15.6-2.2,-1.3242ptUpright (90 degree)	249	49.3
**What type of formula do you use for your child with CLP?**		
Regular formula	440	87.1
29-2.2,-1.3242ptThick formula	65	12.9
**Do you use special feeder/prostheses while feeding your**		
**child with CLP?**		
No	186	36.8
Yes	319	63.2

Table 4 summarizes parental perceptions of medical services. While most parents received adequate information, 70.3% had to travel for medical appointments, with 33.5% traveling for over an hour. School leave for medical visits was generally easy to obtain, though 13.1% found it challenging to obtain. Work-related missed appointments were reported by 37.4%, and 20.2% missed appointments due to a lack of childcare. Appointment waiting times ranged from 1–6 days (28.7%) to over 7 weeks (24.8%).

**Table 4:** Descriptive analysis of parental perceptions about their CLP children's medical services and appointments.

**Variable**	**Frequency**	**Percentage**
**Do you think you have been provided with enough information about CLP from your doctor/the community? mean (SD) - Agreement**		3.68 (1.23)
Completely disagree	42	8.3
Disagree	39	7.7
Neutral	124	24.6
Agree	135	26.7
Completely agree	165	32.7
Do you travel for your appointments?		
No	150	29.7
28.5-2.2,-1.3242ptYes	355	70.3
**How far is the specialized care center that your child with CLP**		
**follows up in from your residence?**		
Very Near	35	6.9
Near	122	24.2
Slightly far away	115	22.8
Quiet Far	120	23.8
28.6-2.2,-1.3242ptVery far	113	22.4
**How much time do you need to get to the specialized care**		
**center where your child with CLP follows up in?**		
< 15 min	26	5.1
15–30 min	112	22.2
40–60 min	97	19.2
60 min	101	20
28.6-2.2,-1.3242pt> 1 h	169	33.5
**How easy/difficult is it to obtain a school leave for your**		
**diseased child's medical appointments?**		
Very easy	160	31.7
Easy	185	36.6
Slightly difficult	75	14.9
Quiet difficult	66	13.1
28.6-2.2,-1.3242ptVery difficult	19	3.8
**How many medical appointments have you missed because of**		
**your work?**		
None	316	62.6
One	47	9.3
Two	54	10.7
Three	36	7.1
28.5-2.2,-1.3242ptFour or more	52	10.3
**How many medical appointments have you missed because**		
**of your child's school?**		
None	402	79.6
One	41	8.1
Two	30	5.9
Three	19	3.8
28-2.2,-1.3242ptFour or more	13	2.6
**How many medical appointments have you missed because**		
**you could not find caregiver for your other children?**		
None	403	79.8
One	40	7.9
Two	22	4.4
Three	15	3
28.6-2.2,-1.3242ptFour or more	25	5
**What is the average waiting time for your physician's**		
**appointment?**		
1–6 days	145	28.7
1–2 weeks	145	28.7
3–6 weeks	90	17.8
28.6-2.2,-1.3242pt≥ 7 weeks	125	24.8
**How long is the interval between two consecutive**		
**consultations?**		
At patient convenience	70	13.9
1-3 weeks	158	31.3
4-6 weeks	151	29.9
≥ 7 weeks	126	25

Table 5 presents the overall parental perceptions. The average knowledge score was 20.36/28 (SD = 5.84). Perceived difficulties in travel (7.55/11), missed appointments (4.01/20), and access to medical services (5.05/8) varied significantly.

**Table 5:** Descriptive analysis of the parents' overall perceptions and knowledge about CLP.

**Variable**	**Maximum possible score**	**Mean**	**SD**
Total CLP Knowledge score (0–28 points)	0–28 points	20.36	5.84
Mean perceived Travel difficulty to attend the CLP appointments score	3–11 points	7.55	2.61
Mean perceived Missed CLP medical appointments score	4–20 points	4.01	3.2
Mean Perceived Difficulty having access to CLP treating physicians	4–8 points	5.05	1.88

Regional comparisons revealed significant demographic and socioeconomic differences affecting parental knowledge and access to care. Table 6 compares findings across regions. One-way ANOVA showed significant differences in child age, with Middle Eastern children being older than those from the Gulf and Pakistan (*p* < 0.001). Gulf-region mothers were significantly older than those from the Middle East and Pakistan (*p* = 0.034, *p* = 0.001). Fathers from the Gulf were considerably older than fathers from other regions (*p* < 0.050). Socioeconomic status (SES) varied significantly by region, with Middle Eastern parents having lower SES scores than Gulf parents (*p* < 0.001) and Pakistani parents (*p* < 0.001). No significant correlation was found between family history of CLP and region (*p* = 0.251), nor between right-side CLP prevalence and region (*p* = 0.185). However, Gulf-region parents were more likely to report complete left-sided CLP. Regression analysis revealed that parental knowledge of CLP was significantly lower among parents from Middle Eastern countries and Pakistan than among those from the Arab Gulf region (*p* = 0.015 and *p* = 0.011, respectively). Higher SES was associated with higher knowledge (β = 1.465, *p* < 0.001), as were a family history of CLP (β = 1.428, *p* = 0.009), prenatal diagnosis (β = 1.875, *p* = 0.001), and associated anomalies (β = 1.198, *p* < 0.001). Parents who relied on hospital staff (β = −1.315, *p* = 0.016) or family members (β = −3.041, *p* < 0.001) for information had lower knowledge scores. Feeding practices, such as the use of thickened formulas, were positively correlated with knowledge *(*β = 1.559, *p* = 0.024), whereas upright feeding positions were associated with lower knowledge (β = −0.885, *p* = 0.007). Missed appointments negatively impacted knowledge (β = −0.193, *p* = 0.013), while travel difficulties and access issues were not significant predictors.

**Table 6:** Bivariate analysis of parents measured sociodemographic factors and perceptions about CLP across different regions.

**Variable**	**Arab Gulf countries**	**Middle East**	**Pakistan**	**Test statistic**	***P-value***
	***N*** = **156**	***N*** = **246**	***N*** = **103**		
Affected child age (years), mean (SD)	4.044 (3.83)	4.5 (3.19)	3.77 (4.64)	F_(2, 502)_ = 3.166	0.043
Mother's age (years), mean (SD)	34.60 (7.33)	32.93 (7.21)	30.04 (7.19)	F_(2, 502)_ = 12.3	< 0.001
Father's age (years), mean (SD)	39.09 (7.74)	38.70 (8.39)	34.11 (7.25)	F_(2, 502)_ = 14.90	< 0.001
15.6-2.2,-9.5498ptHouseholds socioeconomic (SES) Index, mean (SD)	0.655 (0.85)	−0.534 (0.83)	0.282 (0.88)	F_(2, 502)_ = 101.41	< 0.001
**Do you have any other family member with CLP?**					
No	111 (71.2)	193 (78.5)	78 (75.7)	*χ2*(2) = 2.75	0.251
15.6-2.2,-1.3498ptYes	45 (28.8)	53 (21.5)	25 (24.3)		
**Child's right side CLP type**					
Not affected	68 (43.6)	125 (50.8)	58 (56.3)	*χ2*(4) = 6.20	0.185
Incomplete	33 (21.2)	55 (22.4)	16 (15.5)		
15.6-2.2,-1.3498ptComplete	55 (35.3)	66 (26.8)	29 (28.2)		
**Child's left side CLP type**					
Not affected	52 (33.3)	120 (48.8)	60 (58.3)	*χ2*(4) = 24.81	< 0.001
Incomplete	34 (21.8)	63(25.6)	17 (16.5)		
15.6-2.2,-1.3498ptComplete	70 (44.9)	63 (25.6)	26 (25.2)		
**What type of cleft palate does your child have?**					
Not affected	22 (14.1)	87 (35.4)	56 (54.4)	*χ2*(4) = 50.3	< 0.001
Incomplete	48 (30.8)	70 (28.5)	22 (21.4)		
15.6-2.2,-1.3498ptComplete	86 (55.1)	89 (36.2)	25 (24.3)		
**Was the cleft diagnosed during pregnancy?**					
No	112 (71.8)	193 (78.5)	92 (89.3)	χ2(2) = 11.24	0.003
15.6-2.2,-1.3498ptYes	44 (28.2)	53 (21.5)	11 (10.7)		
**Does the child have any associated anomalies?**					
No	102 (65.4)	213 (86.6)	26 (25.2)	*χ2*(4) = 126.52	< 0.001
I don't know	14 (9)	4 (1.6)	17 (16.5)		
15.6-2.2,-1.3498ptYes	40 (25.6)	29 (11.8)	60 (58.3)		
**If the child had associated anomalies, what are they?**					
Congenital cardiac disability	18 (11.5)	13 (5.3)	9 (8.7)	*χ2*(2) = 5.24	0.073
Hearing disability	20 (12.8)	15 (6.1)	25 (24.3)	*χ2*(2) = 23.10	< 0.001
Cognitive disability	6 (3.8)	4 (1.6)	16 (15.5)	*χ2*(2) = 29.50	< 0.001
Mental disability	3 (1.9)	8 (3.3)	20 (19.4)	*χ2*(2) = 39.90	< 0.001
Other problems	10 (6.4)	1 (0.4)	0	*χ2*(2) = 18.65	< 0.001
Speech difficulty	2 (1.3)	0	0	*χ2*(2) = 4.72	0.095
Dental problems	2 (1.3)	1 (0.4)	0	*χ2*(2) = 2.33	0.312
15.6-2.2,-1.3498ptDifficulty breathing	0	0	4 (3.9)	*χ2*(2) = 12.84	0.002
**What is/are your sources of information about CLP?** ***N*** = **500**					
Internet	46 (70.5)	138 (56.1)	18 (17.5)	*χ2*(2) = 55.52	< 0.001
Social media	31 (19.9)	55 (22.4)	17 (16.5)	*χ2*(2) = 1.57	0.456
Hospital	72 (46.2)	151 (61.4)	45 (43.7)	*χ2*(2) = 13.5	0.001
Family member	9 (5.8)	19 (7.7)	18 (17.5)	*χ2*(2) = 11.4	0.003
School	0	3 (1.2)	8 (7.8)	*χ2*(2) = 17.30	< 0.001
15.6-2.2,-1.3498ptCausal meeting	1 (0.6)	5 (2)	5 (4.9)	*χ2*(2) = 4.99	0.083
**When was the first time you heard about CLP?**					
During the pregnancy	49 (31.4)	38 (15.4)	42 (40.8)	*χ2*(4) = 42.02	< 0.001
During the pregnancy	28 (17.9)	22 (8.9)	13 (12.6)		
After having a child with CLP	79 (50.6)	186 (75.6)	48 (46.6)		

Parents from the Middle East (β = 1.169, *p* < 0.001) and the Arab Gulf (β = 2.607, *p* < 0.001) reported greater difficulty accessing CLP care than Pakistani parents. SES was not a significant factor (*p* = 0.398). Still, having a child with associated anomalies (β = 0.252, *p* = 0.010) and using the internet (β = 0.616, *p* = 0.001) or hospital staff (β = 0.448, *p* = 0.008) for information were associated with greater access difficulties. Travel challenges (β = 0.084, *p* = 0.003) and frequent missed appointments (β = 0.092, *p* < 0.001) were strong predictors of access difficulties, while higher parental knowledge was associated with fewer perceived barriers (β = −0.030, *p* = 0.029). Analysis of missed appointments revealed that SES was positively correlated with appointment missed rates, with wealthier and more educated parents missing more appointments (*p* = 0.001). Parents in the Arab Gulf and the Middle East had lower missed-appointment rates than parents in Pakistan (*p* < 0.001). Diagnosis type also influenced missed appointments, as parents of children with right cleft lip had higher missed appointment rates (*p* = 0.001), while those with left cleft palate had lower rates (*p* = 0.017). Travel difficulties (*p* < 0.001) and challenges in accessing physicians (*p* < 0.001) were strong predictors of missed appointments, but knowledge of CLP did not significantly impact missed appointments (*p* = 0.170).

Finally, perceived difficulty in attending appointments was significantly associated with cleft type. Parents of children with complete *(p* < 0.001) or partial (*p* = 0.008) cleft palates reported greater difficulties. Difficulties in accessing services were strongly correlated with challenges in attending appointments (*p* = 0.007), and missed appointments further exacerbated these challenges (*p* < 0.001). However, parental knowledge of CLP did not significantly impact perceived appointment difficulties (*p* = 0.409). These results highlight the importance of socioeconomic factors, information sources, and healthcare access in shaping parental knowledge and perceived barriers to CLP care.

## 4 Discussion

CLP is a common congenital condition requiring complex, long-term, multidisciplinary care. Despite the prevalence of CLP, limited data existed on how parental SES, access to healthcare services, and the challenges faced influence parental understanding, practices, and perceptions across different countries, particularly in underrepresented regions such as the Middle East, the Arab Gulf, and South Asia. Therefore, there was a pressing need to conduct the study and provide region-specific data on CLP parental awareness and barriers to care, as well as to evaluate the real-world implications of travel burden, missed appointments, and information sources on care continuity and parental confidence in managing CLP.

While most recognized surgery as a primary treatment and the importance of multidisciplinary care, significant gaps existed in understanding syndromic associations, preventive measures, and genetic or environmental risk factors. Research on parental knowledge of CLP highlights the importance of education and family support. Studies show that combined education significantly improves parents' knowledge and care performance (14). Parents value interventions such as repetition of information, visual aids, and anticipatory guidance (15). However, parents often desire more written and visual information, contact with other CLP families, and better-informed healthcare professionals outside specialized centers (15, 16). SES may influence access to information and treatment (7). Education about stigma and discrimination can significantly improve parents' understanding (17). Children with CLP acquire oral health knowledge through everyday routines, with parents playing a crucial role in maintaining good practices (18).

Regarding parental SES and CLP, the literature reveals complex relationships. Parental diagnosis of CLP influences adaptation, with affected parents reporting more guilt but less anxiety than unaffected parents and later presentation for treatment (19–21). Parents from lower SES backgrounds report more barriers to comprehensive care, and their children have poorer oral health-related quality of life (22, 23).

SES impacts parental psychosocial wellbeing, with lower-income parents experiencing lower self-esteem and perceived social support (24). However, some studies found no significant association between SES and CLP risk (25). Rural families tend to have lower SES and higher rates of complete clefting (26). These findings highlight the importance of considering SES in CLP care and research.

Despite generally favorable perceptions of the adequacy of the information provided, most parents reported the need to travel for specialized care, with over one-third traveling more than 1 h. Difficulty obtaining school leave and missing appointments due to work or lack of childcare were common challenges. These findings emphasize systemic barriers that can disrupt care continuity, especially for families with limited flexibility or support networks. Travel burden and healthcare access challenges were particularly marked among parents from the Middle East and the Arab Gulf compared to those from Pakistan. Parents often experience emotional distress and face public stigma (27). Barriers to care, including financial difficulties, lack of information, and transportation issues, negatively impact outcomes and increase the desire for revision surgeries (28, 29). Delayed surgical repairs are common, even in countries with free comprehensive care (30). Caregivers report unmet support needs, particularly regarding individualized information and service coordination (31). SES influences perceptions of barriers, with lower-income families experiencing greater difficulties (22). Despite these challenges, most caregivers have high expectations for surgical outcomes and report positive reactions post-surgery (32). Improving access to care, enhancing public awareness, and providing better support services are crucial for optimizing outcomes for CLP patients (33). This apparent paradox, in which parents from the Middle East and the Arab Gulf reported greater difficulty accessing CLP services but lower missed-appointment rates than those from Pakistan, likely reflects fundamental differences in healthcare organization and socioeconomic structure. In the Middle East and Gulf regions, care is centralized mainly in tertiary hospitals, which increases travel burden and perceived difficulty in access. However, once integrated into these publicly funded, highly coordinated programs, families are supported by structured follow-up schedules and reminder systems, leading to better appointment adherence. In contrast, in Pakistan, access to care may initially appear easier due to the availability of multiple entry points through private or charitable centers. Yet, long-term continuity is hindered by financial constraints, travel costs, and limited follow-up mechanisms.

Missed appointments were prevalent across all SES levels. Individuals of higher SES were shown to miss appointments due to work-related issues, coinciding with Cassell et al. (34), who found that caregivers recognized that taking time off work impacted seeking care.

Another limiting factor for missed appointments recognized in the data was the geographical location, with parents often traveling more than one hour to reach care, which is in line with the literature, as mentioned by a study published in Saudi Arabia by Allaf et al. (35), parents of lower SES may also rely on public transportation to cross long distances. Additionally, frequent hospital visits add further strain on these families (27). Moreover, missed appointments complicate care, especially with the multidisciplinary care required to reduce morbidity and mortality (4).

Regarding feeding practices, most children were breastfed, and a small percentage relied on mixed feeding, which is in line with the current literature. Mothers initially attempted to breastfeed their children; however, after facing issues such as difficulty latching or creating adequate suction, feeding was shifted to formula (36–38). Along with formula feeding, most parents relied on feeding prostheses to make the process. Goyal et al. found that initially, spoon-feeding was the most utilized method, but it was replaced by bottle-feeding in older infants (39). Another study conducted in Turkey found that infants were initially fed via nasogastric or orogastric tubes and later shifted to palatal obturators (40).

The findings of our study align with a survey conducted in Vietnam, which highlighted the significance of parental knowledge for the quality of care provided to patients, with a greater knowledge gap associated with a greater caregiver burden. Both studies link higher knowledge levels to improved adherence to treatment and better quality of life (41). Barriers to care, such as long travel distances and lower socioeconomic levels, were found to cause a burden on delivering care, which coincides with a study focusing on pediatric orthopedic challenges, the latter research implementing methods to bridge the gap between urban and rural care by utilizing surgical teams in the rural regions and telemedicine, which enhances continuity of care (42).

The findings have important implications for public health policy and clinical practice. Targeted educational interventions are needed, especially for lower SES groups and regions with limited access to specialized care. Early parental education, initiated at or before diagnosis, should emphasize feeding practices, treatment pathways, and available support resources. Enhancing prenatal detection and strengthening referral systems to multidisciplinary cleft teams can ensure timely and coordinated care. Efforts to improve transportation options, expand regional outreach and telemedicine services, and establish community-based follow-up clinics may alleviate access burdens.

Additionally, integrating genetic counseling into antenatal and preconception care could help families with a history of CLP understand risk factors and preventive measures. Enhancing the clarity and consistency of health communication, particularly from hospital staff, can help correct misinformation and improve parental confidence. Future research should assess the impact of these interventions on parental knowledge, adherence, and long-term outcomes in affected children.

The study faced several limitations. First, it relies on self-reported data, which may be affected by recall bias or social desirability bias, potentially leading to inaccuracies. Additionally, the online survey format may limit participation to parents with internet access and digital literacy, introducing selection bias. The generalizability of the findings is also a concern, as the study was conducted within specific geographic and cultural contexts that may not fully represent all parents of children with CLP. While the sample size calculation ensures statistical power, the relatively small number of participants per country may not capture the full diversity of experiences, particularly in regions with significant cultural and socioeconomic variations. Furthermore, the study's cross-sectional design provides only a snapshot of parental knowledge and perceptions at a single point in time, limiting the ability to assess changes over time. There is also a potential response bias, as participants in the study may already have an interest in or awareness of CLP, leading to an overestimation of knowledge in the broader population.

Additionally, despite professional translation and review, minor differences in language interpretation between the Arabic and English versions of the questionnaire may influence responses. Lastly, unmeasured confounding factors, such as the severity of the child's condition, prior medical consultations, and parental education regarding other medical conditions, may affect responses but are not explicitly controlled for in this study. Despite these limitations, the study provides valuable insights into parental perspectives on CLP and identifies key areas for future research and intervention.

## 5 Conclusion

This study reveals regional and socioeconomic disparities in parental knowledge and access to care for children with CLP. Parents from the Arab Gulf and higher socioeconomic groups had better awareness and fewer care barriers, while those from the Middle East and South Asia faced more challenges. Despite moderate overall knowledge, key gaps persist in understanding risk factors and feeding practices. Parental knowledge did not significantly affect access to care, highlighting systemic barriers. The study calls for region-specific education, better health communication, and stronger infrastructure to improve care equity and parental support for children with CLP.

## Data Availability

The original contributions presented in the study are included in the article/supplementary material, further inquiries can be directed to the corresponding author.
